# Gut microbiota and therapeutic approaches for dysbiosis in irritable bowel syndrome: recent developments and future perspectives

**DOI:** 10.3906/sag-2002-57

**Published:** 2020-11-03

**Authors:** Hanna FJELDHEIM DALE, Gülen ARSLAN LIED

**Affiliations:** 1 Department of Clinical Medicine, Centre for Nutrition, University of Bergen, Bergen Norway; 2 Department of Gastroenterology, Department of Medicine, Haukeland University Hospital, Bergen Norway; 3 Department of National Centre of Functional Gastrointestinal Disorders, Haukeland University Hospital, Bergen Norway

**Keywords:** Dysbiosis, gut microbiota manipulation, FODMAPs, probiotics, antibiotics, fecal microbiota transplantation

## Abstract

Increased knowledge regarding the implications of gut microbiota in irritable bowel syndrome (IBS) suggests that a disturbed intestinal microenvironment (dysbiosis) might promote the development and maintenance of IBS symptoms and affects several pathways in the pathology of this multifactorial disease. Accordingly, manipulation of the gut microbiota in order to improve IBS symptoms has evolved as a novel treatment strategy in the last decade. Several different approaches have been investigated in order to improve the gut microbiota composition. Dietary modifications including supplementation with fibers, prebiotics, and probiotics are shown to improve symptoms and composition of gut microbiota in IBS; however, the exact probiotic mixture beneficial for each individual remains to be identified. The use of antibiotics still needs confirmation, although promising results have been reported with use of rifaximin. Fecal microbiota transplantation (FMT) has recently gained a lot of attention, and several placebo-controlled trials investigating FMT obtain promising results regarding symptom reduction and gut microbiota manipulation in IBS. However, more data regarding long-term effects are needed before FMT can be integrated as a customized treatment for IBS in the clinical routine.

## 1. Introduction

Irritable bowel syndrome (IBS) is a common functional gastrointestinal disorder (FGID), affecting between 10% and 20% of the population globally [1]. The condition is characterized by a combination of symptoms including abdominal pain, bloating, distention, flatulence, and disturbed bowel habits seen as constipation (IBS-C), diarrhea (IBS-D), or a combination of both (IBS-mixed) [2]. Currently, the Rome IV Diagnostic Criteria, providing symptom-based criteria for diagnosis, is applied for the diagnosis of IBS and its subtypes [2]. Besides the gastrointestinal (GI) symptoms, most patients suffering from IBS also experience a broad spectrum of extraintestinal symptoms, such as fatigue, fibromyalgia, poor social functioning, and reduced emotional well-being. Thus, although not being a fatal and organic disease, IBS is shown to have severe impact on quality of life [3,4]. 

**Figure 1:**
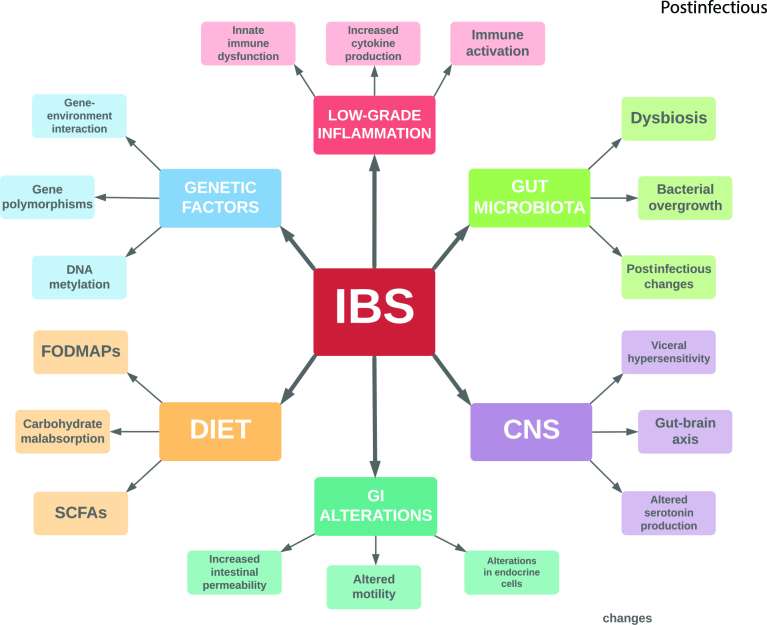
An overview of suggested mechanisms and factors involved in irritable bowel syndrome (IBS).

The etiology of IBS is not fully understood, but it is evident that the condition involves a dysfunction in the gut microbiota, seen as a dysbiosis leading to altered composition and diversity of the microbes in the gut [5]. The increased risk of developing IBS that has been observed after an infectious enteritis and/or excessive use of antibiotics supports the assumption of a dysbiotic gut as a contributor to the onset of IBS symptoms [6,7]. In addition to altered gut microbiota, alterations in one or more of the control systems that contributes to the regulation of bowel function, including the central nervous system, the enteric nervous system, the enteroendocrine system, and the enteric immune system is presumed to be involved in the generation of IBS [8,9]. Suggested mechanisms includes alterations in the gut-brain axis, low-grade inflammation, visceral hypersensitivity, abnormalities in the GI endocrine cells, changes in the GI motility, postinfectious changes, bacterial overgrowth, malabsorption of carbohydrates, abnormalities in serotonin metabolism, gene interactions, and alterations in the gut microbiota composition [3,5,6,10,11]. An overview of suggested mechanisms and factors involved in the pathophysiology of IBS is summarized in Figure 1. 

As the gut microbiota composition and activity is decisive for several pathways suggested to be involved in IBS, different therapeutic approaches aim to target and manipulate a potentially dysbiotic gut. The current review is an attempt to summarize the most recent developments in treatment options targeting the gut microbiota and point out future perspectives for novel treatment approaches in IBS related to disturbed gut microbiota.

## 2. Microbiota and IBS

### 2.1. The human gut microbiota 

The human gut is inhabited by huge amounts of microorganisms important for maintaining GI homeostasis, including bacteria, virus, fungi, archae, and eukaryotes. More than 2000 different bacterial species have been identified in the human gut, of whom the majority belongs to the four main phyla *Bacteroidetes* , *Firmicutes* , *Actinobacteria* , and *Proteobacteri* [12]. While the gut microbiota is the present community of microorganisms, the gut microbiome refers to the entire set of genomic elements decisive for the microbiota in the gut. A healthy composition of the gut microbiome is essential for a broad range of physiological functions, as the gut microbial genes are decisive for the bacterial richness in the gut and hence the function of the gut microbiota [13]. In healthy subjects, the composition of the gut microbiota is determined by both genetic and environmental factors, of which the genetic factors are suggested to explain only 5–10% of the bacterial variability among individuals [14]. The environmental factors decisive for the gut microbiota composition include diet, geographical location, surgery, smoking, depression, frequency of treatment with antibiotics and use of certain nonantibiotic drugs, among others [15]. Microbial richness, seen as bacterial diversity, is usually considered an indicator of good health. In contrast, reduced bacterial diversity and imbalance of the gut microbiota, referred to as dysbiosis, has been associated with impaired metabolism and a broad range of different diseases [16].

### 2.2. Implications of the gut microbiota in IBS

It is suggested in the literature that the gut microbiota profile of IBS patients differs from the gut microbiota profile of healthy controls [17–20]. In addition, several differences in gut microbiota composition among IBS subtypes have been detected [21]. Methane-producing bacteria, such as *Methanobacterialles* , have been reported to be more abundant in IBS-C and less abundant in IBS-D compared to healthy subjects, whereas butyrate-producing bacteria, such as *Erysipelotrichaceae* and *Ruminococcaceae* , are found in lower concentration in IBS patients than in healthy subjects [22]. Although the findings are overall inconsistent regarding gut microbiota composition and diversity in IBS, data in general suggest that there is a relative abundance of proinflammatory bacterial species such as *Enterobacteriaceae* , and decreased presence of *Lactobacilli* and *Bifidobacteri* [20]. In addition, an increase in *Firmicutes* to *Bacteroidetes* ratio, and increased *Streptococci* and *Ruminococcus* species are observed when IBS patients are compared to healthy subjects [23–26]. 

IBS patients are most often characterized by a reduction in bacterial diversity, referred to as dysbiosis, when compared to healthy subjects [19]. Of note, it is suggested that IBS patients who do not respond to dietary interventions suffer from a severe dysbiosis [15]. However, although it is well documented that IBS patients have alterations in the gut microbiota composition and can be characterized by a dysbiotic gut, the specific microbial signature characterizing these patients is still unknown [5].

## 3. Current therapeutic options targeting gut microbiota in IBS 

A multidisciplinary approach is the recommended treatment option in IBS [2]. A better understanding of the disease, combined with life-style interventions including diet, exercise, and breathing techniques, is proven to be effective for reducing symptom severity in IBS patients [3]. In addition, psychological and behavioral treatment and hypnotherapy has also been shown to reduce IBS symptoms and improve quality of life [27]. Nonpharmacological treatment options are preferably the first-choice in IBS, as available drugs are most likely to target only the predominant symptom. Due to the broad diversity of symptoms experienced by most IBS patients, drug treatment is often perceived as inadequate and do not lead to total control of symptoms [2]. Hence, the need of novel and more personalized treatment approaches in IBS is of great need. 

### 3.1. Dietary strategies modulating gut microbiota in IBS 

Diet composition and food intake have been shown to play an important role in the symptom generation in IBS patients, and most of them claim that their symptoms can be related to the consumption of different foods [28]. In recent years, the interaction between diet, gut microbiota composition, and severity of IBS symptoms has gained a lot of attention and emerged as an important pathophysiological basis for the treatment [29]. Lately, the “brain-gut” axis is referred to as the “brain-gut-microbiota” axis [30]. Diet contributes in the pathogenesis of IBS by modulating the gut microbiome and the normal gut microenvironment [31]. The food we eat and its breakdown products are capable of altering the gut microbiota composition and the colonic fermentation, in addition to interacting with the gut immune system and affecting the gut permeability, motility, and visceral sensation [32]. 

#### 3.1.1. Basic dietary advices in IBS 

Several modifications in the diet are shown to enhance symptom severity in IBS. The first line dietary treatment for IBS patients is to follow the dietary guidelines from the modified National Institute for Health and Care Excellence (NICE) diet, as recommended by the British Dietetic Association [33]. The NICE-diet highlights the importance of regular meals and recommends reducing the intake of carbonated drinks, chewing gum, fatty and spicy foods, coffee, alcohol, onion, cabbage, and beans as well as supplementing the diet with fibers such as psyllium husk. 

#### 3.1.2. Dietary fiber modifications

Dietary fibers can be divided into soluble and insoluble fibers, according to their chemical and physiological properties. Soluble fibers can further be divided into viscous (gel-forming) and nonviscous fibers [34]. The dietary fibers include nondigestible short-chain and long-chain carbohydrates and lignin, a complex polymer percent in plants [35]. Overall, including fibers in the diet has several beneficial health effects, such as improving stool consistency, lowering blood cholesterol levels, and improving glycemic control [36]. For a long time, it was believed that IBS was caused by a deficient intake of fibers; hence, it was widely recommended that IBS patients increased their intake [37]. However, the effects of different fibers have proven to be of importance, as soluble fibers are shown to improve IBS symptoms while insoluble fibers, on the other hand, are shown to exacerbate symptoms [38,39]. Supplementation with soluble and moderately fermentable fibers such as psyllium and oats is a recommended treatment in IBS, shown to improve symptom severity and beneficially modulate stool consistency in all IBS-subtypes [36]. Several randomized controlled trials (RCTs) investigating the effect of fiber supplementation on microbiota composition in healthy subjects have suggested that supplementation with fructo-oligosaccharides (FOS) and galacto-oligosaccharides (GOS), known for their prebiotic activity, increases the abundance of *Bifidobacterium* , an oligosaccharide fermenting bacteria suggested to be beneficial for human health [40]. 

A broad range of studies have investigated the effect of supplementation with different types of fibers in patients with IBS, and increasing amount of evidence indicates that the dietary fibers acting as prebiotics, such as inulin and oligosaccharides, are able to influence the composition of the gut microbiota [41–43]. Fermentation of dietary fibers by colonic bacteria leads to the production of short-chain fatty acids (SCFAs), and concentrations of fecal SCFAs have been investigated as a marker of fecal fermentation reflecting the gut microbiota activity in IBS patients [44]. Distinct alterations in fecal SCFA concentrations have been reported between IBS patients and healthy controls, and propionate and butyrate in particular have been suggested as possible biomarkers for the distinction between IBS and healthy individuals [45]. 

#### 3.1.3. The low-FODMAP diet

If basic dietary modifications do not improve symptoms, a diet with a restricted intake of fermentable oligosaccharides, disaccharides, monosaccharides, and polyols (FODMAPs) is proven by several metaanalyses to significantly reduce symptom severity in IBS, especially abdominal pain and bloating [46,47]. FODMAPs are short-chained carbohydrates that are poorly absorbed in the small intestines, and when fermented by colonic bacteria, production of gas and osmotic action causes symptoms such as bloating and diarrhea [47]. Notably, a diet with a restricted intake of FODMAPs is associated with distinct alterations in both function and composition of the gut microbiota, as well as modulations in fecal fermentation and production of SCFAs [44,48]. 

In a recent publication, we reported on the effect of a low-FODMAP diet combined with supplementation with FOS or placebo, in 20 patients with IBS-D or IBS-M [42]. We found the low-FODMAP diet to overall consistently improve symptom severity, and significantly more patients reported symptom relief when supplemented with placebo (maltodextrin) for 10 days, compared to the FOS supplementation (80% vs. 30%). The serum levels of proinflammatory interleukins (IL-6 and IL-8), total concentrations of SCFAs and n-butyric acid, as well as levels of bacteria in feces ( *Actinobacteria* , *Bifidobacterium* , and *Faecalibacterium prausnitzii* ), significantly decreased in response to the low-FODMAP diet. Ten days of supplementation with FOS increased the levels of these bacteria, whereas the levels of inflammatory markers and concentrations of SCFAs did not change [42]. Accordingly, the long-term health effect of adherence to the low-FODMAP diet as a dietary intervention is not established. The health effects of the changes observed in the gut microbiota composition and fecal fermentation in response to a diet with low content of fermentable carbohydrates is currently unknown [49]; thus, the long-term use of a strict low-FODMAP diet can be questioned. 

Although FODMAPs can be experienced as symptom generators in IBS, most FODMAPs can be classified as prebiotics and serve as food for the gut microbiota [42]. A reduction of FODMAPs may in most cases correlate with a reduction both in dietary fibers and prebiotics, which in the long term might not be beneficial for the gut microbiota composition and activity. Hence, there is a need for establishing the long-term effect of a restricted FODMAP diet on the gut microbiota, as well as for continuing to highlight the importance of reintroducing all those well-tolerated FODMAPs in the diet as soon as possible.

### 3.2. Probiotic supplementation and gut microbiota in IBS

Probiotics are defined as “live microorganisms that, when administered in adequate amounts, confer a health benefit on the host” [50]. Probiotics normally contains gut-friendly bacteria, and sometimes also yeast, and can be provided in the form of a dietary supplement or consumed as ingredients in fermented foods such as yoghurt, kombucha, and sauerkraut. Since an altered gut microbiota composition is suggested as a central contributor to the pathogenesis of IBS, supplementing the diet with probiotics is a well investigated treatment strategy in IBS, which aims to beneficially manipulate a dysbiotic gut [51]. Several metaanalyses of randomized controlled trials (RCTs) investigating the effects of probiotics compared to placebo in IBS patients have concluded that probiotic supplements significantly improve overall IBS symptoms and abdominal pain [52–55]. Of note, the use of monostrain or multistrain probiotics varies between studies, and it is suggested that supplements containing a multistrain probiotic (several different bacterial strains) might be more effective than monostrain supplements in improving symptoms. In a recent review article summarizing the most up-to-date research on the effects of probiotic supplementation on symptom severity in IBS patients, we reported that the effects of probiotic supplementation were more distinct with the use of a multistrain supplement [51]. In addition, we found the effect to be more distinct in the studies with an intervention period of 8 weeks or more, suggesting that probiotics supplemented over a period of time have the potential to improve IBS symptoms [51].

Overall, much remains to be answered as to which strains facilitate the most beneficial effects in IBS. Although probiotic supplements seem to have beneficial effects in improving IBS symptoms, their function and mechanistic effects are relatively unknown. Current literature has suggested that probiotics act through improvement of gut barrier function, inhibition of pathogenic bacteria overgrowth, and prevention of pathogenic invasion of the host, as well as production of signal substances such as SCFAs and neurotransmitters [56]. The fact that the degree of dysbiosis and gut microbiota composition most likely varies greatly both between subtypes of IBS patients and between each subject leads to the question of whether it is possible to target these individual differences in future therapy. According to current literature, individual microbiota profiling may in the future enable personalized or customized probiotic therapy.

### 3.3. Antibiotics in IBS

Nonabsorbable antibiotics have been investigated in the treatment of IBS in several large-scale double-blinded and placebo controlled RCTs, and have been found to serve as an effective treatment option [5]. In 2015, rifaximin, a rifamycine derivate with a broad range of antibacterial effects against aerobic and anaerobic organisms in the GI tract, was approved by the US Food and Drug Administration for the treatment of adults with IBS-D [57]. Less than 0.5% of the oral dose of rifaximin is absorbed; hence, this antibiotic has low toxicity and no significant adverse effects and drug interactions [58]. Although the route of action of nonabsorbable antibiotics in IBS is unclear, relief of IBS symptoms is suggested to occur due to reduction in the total gut bacterial load and changes in the composition of the gut microbiota, as well as modulation of the intestinal permeability and the gut microbiome [20]. 

Three multicenter RCTs, named TARGET 1-3, have demonstrated a beneficial effect of rifaximin in improving IBS symptoms [59,60]. TARGET 1 and 2 included IBS patients without constipation. The primary outcome was proportion of patients with relief of global IBS symptoms, with adequate relief defined as self-reported symptom relief for at least 2 of the first 4 weeks after the intervention. Both interventions revealed that rifaximin given in doses of 550 mg 3 times a day for 2 weeks significantly reduced overall symptoms and bloating when compared to placebo (40.8% vs. 31.2% in TARGET 1 and 40.6% vs. 32.2% in TARGET 2) [59]. The TARGET 3 study included adult patients with only IBS-D, and those patients responding to a 2-week treatment with rifaximin as provided in TARGET 1 and 2 but relapsed during an observation phase, were randomly assigned to an additional treatment with either rifaximin or placebo for another 2 weeks. The percentage of responders were significantly greater with rifaximin group with placebo (38.1% vs. 31.5%), and the rates of adverse events were low [60]. This study demonstrates that repeated treatments with rifaximin are well tolerated and can be considered effective in reducing symptom in patients suffering from diarrhea predominant IBS.

Although rifaximin is not indicated in IBS-C, a small double-blind trial has suggested that rifaximin may improve symptoms also in patients with constipation [61]. The study showed that rifaximin given in combination with neomycin significantly reduced the degree of constipation and bloating, compared to neomycin given with placebo. The constipation severity scores were significantly lower in the neomycin and rifaximin group (28.6 ± 30.8) compared to neomycin alone (61.2 ± 24.1) [61].

## 4. Future perspectives and potential treatments manipulating the gut microbiota in IBS 

### 4.1. Fecal microbiota transplantation in IBS

Fecal microbiota transplantation (FMT) has recently gained a lot of attention due to the strong evidence for the role of dysbiosis in the IBS pathogenesis. FMT is an approach involving application of a solution of fecal material from a healthy donor into the gut of a receiver, aiming to restore a dysfunctional microbial composition into a healthy homeostasis and hence improve the function of the gut microbiota [5]. A Japanese open-label study in 10 IBS patients reported in 2017 that FMT treatment improved both the psychological status and stool form. In addition, the authors suggest that a donor holding a high fecal concentration of *Bifidobacterium* might be a positive predictor for successful FMT [62]. A systematic review by Halkjær et al. reported in 2017 that 28 out of 48 patients (58%) experienced a beneficial outcome of FMT treatment, and importantly, no adverse events were reported [63]. The review included only a restricted number of FMT studies, as the data from RCTs then were limited. In the last two years, several RCTs evaluating the effect of FMT in IBS have been published, reporting somehow conflicting results regarding alleviation of IBS symptoms. 

Three recent Norwegian studies, all using FMT administered through gastroscopy or colonoscopy, have reported beneficial effects. Johnsen et al. assessed the effect of FMT in a parallel-group RCT, with 60 patients assigned to FMT treatment and 30 assigned to placebo [64]. The trial included IBS patients with moderate-to-severe IBS-D or IBS-M, and the primary outcome was reduction in total IBS severity scoring system (IBS-SSS) of more than 75 points. Fifty-five patients assigned to FMT treatment and 28 assigned to placebo completed the trial. They reported that the FMT treatment significantly reduced symptoms compared to placebo, with a 65% response in the FMT group and a 43% response in the placebo group [64]. 

Results from our study, including 13 IBS patients and 13 healthy donors receiving FMT, support the findings of Johnsen et al. [65,66]. According to symptom scores, the symptoms were significantly improved following FMT [65]. When analyzing fecal samples, we found that the donors and the IBS patients had significantly different bacterial strains signals for *Ruminococcus gnavus* , *Actinobacteria* , and *Bifidobacteria* , in addition to significantly differences between several gut microbiota taxa and concentrations of SCFAs at baseline, which of note became nonsignificant 3 weeks following FMT [66]. At 20/28 weeks after FMT, the changes in the gut microbiota were similar between IBS patients and donors; thus, the patient’s microbiota profile became more or less similar to those of the donors. Those IBS patients responding to FMT had different gut microbiota profile and SCFA profile than the nonresponders, and significant correlations were reported between the gut microenvironment and IBS symptoms [66].

El-Salhy et al. recently performed a placebo-controlled RCT, evaluating the effect of two different doses of FMT [67]. This study, including 165 IBS patients allocated to either placebo 30 g or 60 g FMT, demonstrated that FMT was effective in reducing IBS symptoms and fatigue, as well as increasing the quality of life. The primary outcome was a positive response defined as a reduction of 50 points or more in the total IBS-SSS score. Response occurred in 23.6% of the patients in the placebo group, 76.9% in the 30 g FMT group, and 89.1% in the 60 g FMT group, suggesting that the response to FMT increases according to dose. In addition, a significant change in gut bacterial profile was observed in the groups receiving FMT [67]. Following, an open-label study including the 10 patients not responding to the 30 g FMT treatment was conducted [68]. The ten patients received a 60 g FMT transplant, of which 7 (70%) responded to treatment with significant improvements in IBS-symptoms, degree of fatigue, and quality of life. These results further highlight that increasing doses and/or repeated FMT increases the response [68]. 

Results conflicting with those of the Norwegian studies were presented by a Danish trial by Halkjær et al. [69]. This randomized, double-blind, and placebo-controlled trial included 52 patients with moderate-to-severe IBS, randomized to capsules with FMT or placebo for 12 days, with 6 months of follow up. Here, a significant reduction of symptoms according to the IBS-SSS core was observed for placebo, but not for the FMT treatment. The patients receiving the FMT capsules had an increase in the fecal microbiota diversity that was not observed in the placebo group; however, the authors state that altered gut microbiota is not enough to obtain clinical improvement in IBS [69]. Accordingly, although several studies investigating FMT as a treatment option in IBS obtain promising results regarding symptom severity and gut microbiota manipulation, the results are still conflicting. The outcome of FMT seems to be dependent on the donor; hence, a so-called “superdonor” seems to be essential for FMT to be an effective treatment [14]. 

Currently, FMT is only investigated in a research setting, and the available data on potential effects are too limited to draw sufficient conclusions. More RCTs investigating the long-term effects of FMT and its route of administration need to be carried out in order to potentially be able to offer FMT as a standardized treatment approach for IBS in a clinical setting. 

### 4.2. Targeting the gut microbiota in IBS

**Figure 2:**
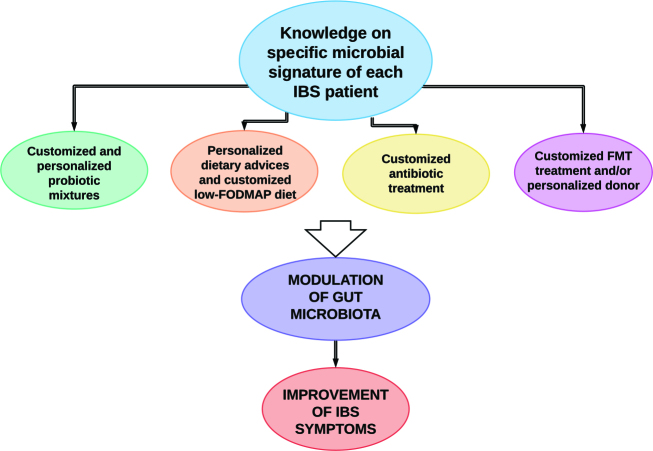
Suggested future therapies for irritable bowel syndrome (IBS), enabled by mapping of the individual gut microbiota profile and use of personalized and targeted treatment strategies.

As our understanding of the implications of the gut microbiota in IBS is constantly evolving, the potential of treatments manipulating both the gut microbiome and the gut microbiota aiming to improve GI symptoms and severity of IBS is widening. Hopefully, determining the exact microbiota profile and degree of dysbiosis in each IBS patient will soon be an available tool for personalized treatment options, such as customized probiotic or FMT treatments and/or targeted use of antibiotics. An overview of some suggested future therapeutic strategies in IBS is shown in Figure 2. 

## 5. Conclusions

There is strong growing evidence suggesting that dysbiosis is central in the pathophysiology of IBS. Manipulation of the gut microbiota in order to improve IBS symptoms has evolved in the last decade as a novel treatment strategy and several different approaches have been investigated in order to improve the gut microbiota composition and hence improve symptoms. Dietary modifications including supplementation with fibers and probiotics are shown to improve symptoms and gut microbiota in IBS, although the exact probiotic mixture beneficial for each individual remains to be identified. The use of antibiotics still needs confirmation, although promising results have been reported with the use of rifaximin. Several studies investigating FMT obtain promising results regarding symptoms and gut microbiota manipulation in IBS; however, more data including long-term effects of FMT are required before it can be used as a customized treatment in the clinic.

## Contribution of authors

Both authors have contributed to the paper. HFD wrote the paper, and GAL provided writing assistance and corrected the manuscript.
